# Faecal haemoglobin concentration and colorectal cancer site, stage and grade in a symptomatic cohort

**DOI:** 10.1111/codi.17187

**Published:** 2024-09-25

**Authors:** Nicholas G. Farkas, James O'Brien, James Norman, Jackie Steinke, Kai Shing Yu, Martin Whyte, Iain Jourdan, Tim Rockall, Sally C. Benton

**Affiliations:** ^1^ Minimal Access Therapy and Training Unit (MATTU) Royal Surrey Hospital NHS Foundation Trust Guildford UK; ^2^ Faculty of Health and Medical Sciences University of Surrey Guildford UK; ^3^ Department of Clinical Biochemistry Royal Surrey County Hospital, Berkshire and Surrey Pathology Services Guildford Surrey UK; ^4^ NHS Bowel Cancer Screening South of England Hub, Berkshire and Surrey Pathology Services Royal Surrey County Hospital Guildford Surrey UK

**Keywords:** colorectal cancer, faecal immunochemical test, grade, site, stage

## Abstract

**Aim:**

Minimal evidence exists regarding faecal immunochemical tests (FITs) for colorectal cancer (CRC) site, stage and grade in symptomatic patients. The primary aim is to determine any association between faecal haemoglobin concentration (f‐Hb) (analysed with OC‐Sensor™ Pledia) and these prognostic factors. The secondary aim is to determine the association between f‐Hb and anaemia, microcytosis and iron deficiency (Hb, mean corpuscular volume [MCV] and ferritin).

**Methodology:**

Symptomatic 2‐week wait CRC patients with FIT were included (July 2019–October 2022). Median f‐Hb and interquartile range according to sex, stage, grade and site (right‐sided, caecum to transverse colon, R‐CRC; left‐sided, splenic flexure to rectum, L‐CRC) were compared using the Mann–Whitney *U* test. Hb, MCV and ferritin were categorized into two groups and the median f‐Hb was compared using the Mann–Whitney *U* test.

**Results:**

In all, 114 patients (57 women, 57 men) were studied; 46 had R‐CRC (f‐Hb = 113 μg Hb/g) and 68 had L‐CRC (f‐Hb = 342 μg Hb/g) (*P* = 0.07). Sixty‐nine were moderately differentiated CRC (f‐Hb = 183 μg Hb/g) and 29 were poorly differentiated (f‐Hb = 866 μg Hb/g) (*P* = 0.04). By T‐stage, 35 were early (T1/2) (f‐Hb = 170 μg Hb/g) and 79 were advanced (T3/4) (f‐Hb = 200 μg Hb/g) (*P* = 0.06). The relationship between f‐Hb and Hb, MCV and ferritin was not significant. Poorly differentiated (*P* = 0.04) and later stage (*P* = 0.02) R‐CRC had significantly lower f‐Hb compared to L‐CRC.

**Conclusions:**

Right‐sided CRC is associated with lower f‐Hb than left. Poorly differentiated and later staged L‐CRC had higher median f‐Hb. These data add to existing evidence suggesting that FIT may be less sensitive for right‐sided CRC. Strategies to mitigate the potential for missed or FIT‐negative right‐sided CRC are required.

**What does this paper add to the literature?**

There is limited understanding of faecal immunochemical testing and colorectal cancer (CRC) site, stage and grade. We demonstrate that right‐sided CRC is associated with lower faecal haemoglobin concentration (f‐Hb) than left. Poorly differentiated, later staged left CRC has higher median f‐Hb. There may be greater potential for right‐sided CRC to be missed.

## INTRODUCTION

Faecal immunochemical tests (FITs) have been validated as a reliable and effective clinical aid in the diagnosis and triage of colorectal cancer (CRC). The high negative predictive value of the FIT at defined thresholds of 2 and 10 μg Hb/g faeces (μg/g) has enabled clinicians to consider using a negative result (<10 μg/g) as a rule out test [1]. There has been limited research into the value of the FIT beyond its primary use as an adjunct for referral or investigation of suspected CRC (e.g., if there is a correlation between faecal haemoglobin concentration [f‐Hb] and tumour features).

No definitive association between f‐Hb and tumour site has been established. Widlak et al. [2] demonstrate that 18 left‐sided CRC and a single high‐grade adenoma had higher median f‐Hb (713 μg/g) compared to six right‐sided CRC (94 μg/g). Hunt et al. [3] published a large retrospective study of duplicate FITs in symptomatic 2‐week wait patients. The authors suggest that the seven patients with CRC and double negative FIT shared similar characteristics with regard to right‐sided tumour site and presence of anaemia. O'Reilly et al. [4] describe higher f‐Hb for screen‐detected left‐sided adenomas, suggesting that FIT has poorer sensitivity for right‐sided neoplasia.

Evidence from the screening population highlights that FIT and tumour stage correlate; a meta‐analysis by Niedermaier et al. [5] reported that FIT sensitivity for Stage I CRC was considerably lower than for other stages, suggesting that later stage tumours are larger and bleed more. Correlation between tumour grade and FIT is more uncertain. Tran et al. [6] reported that missed (interval) CRC in an FIT screening cohort were more likely to be high grade, right‐sided tumours.

Currently, FIT is not used for CRC risk stratification in the UK and remains an adjunct to referral or diagnostic decision making [7, 8]. Recent guidance from the British Society of Gastroenterologists and the Association of Coloproctologists of Great Britain and Ireland states that there is insufficient evidence to recommend the inclusion of FIT in risk prediction scoring [9].

The primary aim was to determine whether a difference exists between f‐Hb and tumour site, stage and grade in a symptomatic cohort. The secondary aim was to determine any association between f‐Hb and anaemia, microcytosis and iron deficiency (measured by haemoglobin [Hb], mean corpuscular volume [MCV] and ferritin).

## METHODS

The CRC database (from a district general hospital in the UK) was accessed and diagnoses from July 2019 to October 2022 were reviewed. The timeframe coincided with the introduction of FITs at our centre. All symptomatic patients with CRC and an f‐Hb result sent at the time of referral were included for analysis. Nationally coordinated bowel cancer screening patients and premalignant lesions were excluded as it was not possible to obtain f‐Hb concentrations for these patients.

Electronic patient records, investigations and results were reviewed. Parameters assessed included sex, CRC site, stage (T stage from TNM classification), grade of cancer, Hb, MCV and ferritin. Tumour site, stage and grade were confirmed from histology (biopsy and/or surgery) and radiology reports. All laboratory investigations included were from within 4 weeks of CRC diagnosis.

All FITs were undertaken with an OC‐Auto Sampling Bottle (Eiken Chemical Co. Ltd, Tokyo, Japan) and analysed on an OC‐Sensor™ Pledia analyser (Eiken Chemical Co. Ltd). The limit of detection is 1 μg/g and the limit of quantification is 6 μg/g.

### Statistical analysis

Baseline demographic characteristics and clinical parameters of 114 CRC patients were analysed (Table 1). Continuously reported variables are reported as median, range and interquartile range. For categorical variables, results are reported as number and percentage. Chi‐squared was used to analyse differences in the distribution of categorical data. Continuous, non‐parametrically distributed variables were analysed using the Mann–Whitney *U* test. *P* values <0.05 were considered statistically significant. All analyses were conducted using SPSS 22.0 (Chicago, IL, USA).

**TABLE 1:** Baseline cohort characteristics.

Characteristics	*n*	%	f‐Hb (μg Hb/g) (median, range)		IQR	Q1‐Q3	*P* value
Sex							
Male	57	50.0	183.0	(0–36360)	1704	67–1771	0.87
Female	57	50.0	298.0	(0–32717)	2097	44–2141	
Cancer site							
Right	46	40.4	113.0	(7–9529)	1561	36–2597	0.07
Left	68	59.7	342.5	(0–36360)	2030	80–2110	
T staging							
Early stage: T1 and T2	35	30.7	170.0	(10–6949)	667	40–707	0.06
Advanced stage: T3 and T4	79	69.3	332.0	(0–36360)	3398	87–3485	
T1	13	11.4	56.0	(10–1922)	98	18–116	
T2	22	19.3	315.5	(19–6949)	1536	72–1608	
T3	62	54.4	540.0	(0–36360)	3877	88–3965	
T4	17	14.9	112.0	(8–13667)	1113	44–1157	
Differentiation of cancer							
Moderate differentiation	69	70.4	183.0	(0–36360)	1464	39–1503	0.04
Poor differentiation	29	29.6	866.0	(12–32717)	6509	121–6630	
Hb							
Anaemia^a^	53	46.5	185.0	(6–22894)	1826	83–1909	0.81
No anaemia	61	62.2	332.0	(0–36360)	1982	41–2023	
MCV							
Microcytosis	10	8.8	153.0	(24–3962)	1958	77–2035	0.78
No microcytosis	104	91.2	258.0	(0–36360)	1832	54–1886	
Ferritin							
Iron deficient <30 μg/L	49	47.6	605.0	(6–32717)	3738	96–3834	0.18
Not iron deficient >30 μg/L	54	52.4	225.0	(0–36360)	970	56–1026	
Iron deficiency anaemia^a^							
Present	36	35.0	264.0	(6–22894)	3424	92–3516	0.65
Absent	67	65.0	332.0	(0–36360)	1866	56–1922	

*Note*: Grade data were not recorded in 16 patients (because no biopsy was performed), and ferritin was not measured in 12 patients.

Abbreviations: f‐Hb, faecal haemoglobin concentration; Hb, haemoglobin; IQR, interquartile range; MCV, mean corpuscular volume.

^a^ Men <130 g/L; women <120 g/L, ferritin <30 μg/L.

## RESULTS

In all, 114 patients (men 57: women 57) were included.

### Colorectal cancer site

There is a non‐significant association between higher median f‐Hb and left‐sided CRC (342 μg/g) compared to right (113 μg/g, *P* = 0.07).

### Stage

For T1 CRC, median f‐Hb was 56 μg/g. For T2, median f‐Hb = 315 μg/g, T3 = 540 μg/g and T4 = 112 μg/g. When comparing early (T1/2) and late stage (T3/4) CRC, a modest but non‐significant difference in f‐Hb (170 vs. 200 μg/g respectively) was observed.

### Grade

There was a statistically significant higher median f‐Hb in patients with poorly differentiated CRC versus moderately differentiated CRC (866 vs. 183 μg/g; *P* = 0.04).

### Haemoglobin/MCV/iron deficiency

No differences were observed between f‐Hb and anaemia (*P* = 0.81) or microcytosis (*P* = 0.78). Patients with higher f‐Hb were more iron deficient. Iron‐deficient patients had a median f‐Hb of 605 μg/g, whereas non‐iron‐deficient patients had a median f‐Hb of 225 μg/g (*P* = 0.18). No correlation was seen between f‐Hb and Hb (Pearson's correlation coefficient *r* = −0.11, *P* = 0.26), MCV (*r* = −0.17, *P* = 0.07) and serum ferritin (*r* = −0.15, *P* = 0.13).

### Left versus right CRC

The cohort was stratified by anatomical site and, for all variables analysed, left‐sided CRC had higher median f‐Hb (except T2 tumours) as demonstrated in Table 2. For poorly differentiated CRC, left‐sided had significantly higher median f‐Hb versus right‐sided (*P* = 0.04); the same was found for advanced stage CRC (T3/4) (*P* = 0.02). When grade and stage were combined there was a statistically significant difference with higher f‐Hb noted in left‐sided CRC (*P* = 0.02). Right‐sided CRC had lower f‐Hb irrespective of anaemia, microcytosis and iron deficiency.

**TABLE 2:** Comparison of f‐Hb between right‐sided and left‐sided CRC.

	f‐Hb (μg/g)								
	Right				Left				
	Median	Range	IQR	Q1–Q3	Median	Range	IQR	Q1–Q3	*P* value
Differentiation grading									
Moderate differentiation (*n* = 69)	110	(7–9529)	1469	29–1498	279	(0–36360)	1459	56–1515	0.33
Poor differentiation (*n* = 29)	422	(12–8168)	2551	99–2650	3975	(83–32717)	10016	271–10287	0.04
T staging									
Early stage: T1 and T2 (*n* = 35)	63	(14–3800)	1730	24–1754	177	(10–6949)	580	51–631	0.82
Late stage: T3 and T4 (*n* = 79)	114	(7–9529)	1658	39–1697	602	(0–36360)	4503	154–4657	0.02
T1 (*n* = 13)	25	(14–63)	39	15–54	56	(10–1922)	233	28–261	0.33
T2 (*n* = 22)	1398	(34–3800)	2730	225–2955	260	(19–6949)	1085	67–1151	0.28
T3 (*n* = 62)	125	(7–9529)	2345	44–2389	1202	(0–36360)	5107	231–5338	0.02
T4 (*n* = 17)	103	(8–2267)	776	32–808	175	(26–13667)	3020	74–3094	0.36
Hb									
Anaemia (*n* = 53)	125	(8–9529)	1748	48–1796	271	(6–22894)	1925	122–2047	0.16
No anaemia (*n* = 61)	52	(7–9315)	1030	19.25–1049	352	(0–36360)	3429	56–3485	0.10
MCV									
Microcytosis (*n* = 10)	121	(24–3962)	1808	87–1895	271	(48–2457)	NA	NA	0.83
No microcytosis (*n* = 104)	112	(7–9529)	1464	34–1498	352	(0–36360)	1966	81–2047	0.08
Ferritin									
Iron deficient <30 μg/L (*n* = 49)	422	(8–9529)	3778	39–3817	605	(6–32717)	6789	160–6949	0.28
Not iron deficient >30 μg/L (*n* = 54)	54	(7–683)	97	24–121	352	(0–36360)	1763	88–1850	0.001
Iron deficiency anaemia									
Present (*n* = 36)	200	(8–9529)	3831	38–3869	271	(6–22894)	4570	133–4703	0.58
Absent (*n* = 67)	76	(7–9315)	556	31–586	412	(0–36360)	3392	93–3485	0.01

	f‐Hb (μg/g)								
	Right (*n* = 46)				Left (*n* = 68)				
	Median	Range	IQR	Q1–Q3	Median	Range	IQR	Q1–Q3	*P* value
Poorly differentiated, early stage (*n* = 2)	—	—	—		—	—	—		—
Poorly differentiated, advanced stage (*n* = 27)	200	(12–8168)	1986	95–2081	4827	(83–32717)	10787	345–11132	0.02
Moderately differentiated, early stage (*n* = 30)	49	(14–2110)	1131	22–1152	177	(10–3673)	364	51–415	0.66
Moderately differentiated, advanced stage (*n* = 39)	114	(7–9529)	3837	32–3869	568	(0–36360)	2260	126–2385	0.23

Abbreviations: f‐Hb, faecal haemoglobin concentration; Hb, haemoglobin; IQR, interquartile range; MCV, mean corpuscular volume.

Table 3 outlines seven left‐sided and six right‐sided CRC with negative (*n* = 5) or low positive (*n* = 8) f‐Hb.

**TABLE 3:** Comparison of CRC with f‐Hb <20 μg/g.

Sex	Age	FIT value (μg Hb/g)	Hb (g/dL)	MCV (fL)	Ferritin (μg/L)	Cancer site	Stage	Metastases	Node	Histology
Male	79	<6	145	98.8	68	Sigmoid	T3	M0	N0	Moderately differentiated adenocarcinoma
Female	88	<6	126	91.5		Descending	T3	M0	N0	Moderately differentiated adenocarcinoma
Female	87	6	104	100.2	26	Rectum	T3	M0	N0	Moderately differentiated adenocarcinoma
Male	85	7	140	91.1	50	Transverse	T3	M0	N0	Moderately differentiated adenocarcinoma
Female	74	8	94	91.2	8	Transverse	T4	M1	N1	Poorly differentiated adenocarcinoma
Female	77	10	139	93.1		Rectosigmoid	T1	M0	N0	Moderately differentiated adenocarcinoma
Male	74	12	98	81.3	14	Transverse	T3	M0	N1	Poorly differentiated adenocarcinoma
Female	72	14	142	99.7	19	Ascending	T1	M0	N1	Moderately differentiated adenocarcinoma
Male	76	16	118	91.8		Rectum	T4	M1	Nx	Poorly differentiated adeno‐neuroendocrine carcinoma
Male	59	17	149	96.2		Rectum	T1	M0	Nx	Moderately differentiated adenocarcinoma
Male	72	17	136	93.6	36	Caecum	T3	M0	N0	Moderately differentiated adenocarcinoma
Female	88	17	114	93.4	16	Caecum	T3	M0	N0	Moderately differentiated adenocarcinoma
Male	65	19	139	95.6		Rectum	T2	M0	N1	Moderately differentiated adenocarcinoma

Abbreviations: CRC, colorectal cancer; f‐Hb, faecal haemoglobin concentration; FIT, faecal immunochemical test; Hb, haemoglobin; IQR, interquartile range; MCV, mean corpuscular volume.

## DISCUSSION

Left‐sided CRC has a higher median f‐Hb than right‐sided CRC. This association between CRC site and f‐Hb has been reported elsewhere; Cunin et al. [10] highlight seven FIT‐negative patients, of whom six had caecal adenocarcinomas and five were anaemic. Hunt et al. [3] highlight seven double negative CRC with two negative FITs, of which six were right‐sided. Chapman et al. [11] describe a cohort of 40 patients, where right‐sided tumours had a significantly lower FIT result than left‐sided: median 41.6 (11.2–406.8) versus 286.8 (142–5076.8) μg/g (*P* = 0.03). Our data highlight two FIT‐negative right‐sided and three left‐sided CRC.

It remains unclear why f‐Hb appears to be lower in right‐sided cancers. It has been suggested that degradation of Hb during gastrointestinal transit results in loss of detectable epitopes, reducing the sensitivity of FIT for proximal colonic lesions [2, 12]. Hb from right‐sided tumours will have a longer colonic transit time through the large bowel, resulting in increased mixing with faecal content. This may dilute subtle bleeding from a right‐sided tumour, whereas Hb may be more available on the stool surface in left‐sided tumours. Faecal content of the left colon is bulkier than that of the right, with salt and water absorbed in the ascending and transverse colon. Firmer stool passing the tumour has greater potential to cause trauma and lead to fresh haemorrhage. Variation in FIT sampling technique may capture more faecal content from the outside of stool than the centre [13, 14]. Serial or repeat f‐Hb testing may mitigate some of these concerns and offer a cost‐effective, non‐invasive test that may help minimize the risk of FIT‐negative CRC [3, 15]. A number of studies have shown that repeating FIT increases sensitivity and reduces the rate of missed cancers, even for right‐sided tumours [3, 16].

The overall negative predictive value for f‐Hb has been outlined at population level in a symptomatic cohort. As demonstrated in Table 3, the possibility of a missed cancer diagnosis is ever present, with negative or low f‐Hb occurring in both left‐ and right‐sided CRC equally. Larger studies are needed to determine positive and negative predictive value per anatomical site and help with interpretation of a negative or low positive result.

Stage‐specific sensitivity of FIT for CRC in symptomatic patients is reported in a systematic review and meta‐analysis by Niedermaier et al. [5]. Stage I–IV sensitivity was 79% (Stage I), 88% (II), 85% (III) and 87% (IV). Our data support these findings, highlighting that early stage CRC had lower f‐Hb versus late stage. It is possible that smaller tumours may bleed less frequently and therefore be harder to detect from a single f‐Hb.

Despite the limited cohort size, our results suggest there may be a relationship between poorly differentiated CRC and higher f‐Hb. However, the majority of poorly differentiated tumours (27/29) were also stage T3/T4. The association between higher grade and more advanced stage suggests the observed higher f‐Hb could be related to grade, stage or both. Therefore, there is limited evidence as to whether the f‐Hb can be utilized as a prognostic indicator.

No significant differences were observed relating to anaemia and f‐Hb. Lower ferritin appears associated with higher f‐Hb than MCV or Hb. A low ferritin may precede anaemia and represents an early marker that could be investigated further [17].

Larger studies demonstrate a significant association between anaemia and f‐Hb. Chapman et al. [11] report that f‐Hb was significantly higher in patients with anaemia versus those without (*P* < 0.001). CRC detection was also higher in anaemia (9%) compared to non‐anaemic patients (2.3%). Almilaji et al. [18] compared CRC diagnosed via bowel cancer screening, detection of iron‐deficient anaemia or symptomatology. Anaemia (82.5%) was found to be the only predictor of right‐sided CRC.

Our data support the suggestion that FIT‐negative CRC may occur more commonly in the right‐sided cohort, given the lower median f‐Hb. Reliance on FIT alone could result in missed CRC. Right‐sided CRC presents more commonly with weight loss and microcytic anaemia [19]. As evidenced by other authors, iron deficiency anaemia is a recognized risk factor for CRC and is associated with right CRC [20]. Our data highlight that right‐sided CRC, irrespective of anaemia, microcytosis and iron deficiency, correlated with lower median f‐Hb. Therefore, anaemia, even in the presence of low f‐Hb, could raise clinical suspicion and should potentially trigger investigation.

This study has several limitations, including the relatively small cohort size of 114. Not every patient underwent biopsy, or had ferritin measured, therefore excluding some patients from final analyses. The findings are only applicable to the symptomatic cohort; extrapolation of these data to screen‐detected CRC requires caution. Only adenocarcinomas were analysed, but there is a suggestion that certain tumour types (such as neuroendocrine tumours) are less commonly associated with bleeding [21, 22]. Conversely, f‐Hb is raised in a number of other benign conditions, such as hyperplastic polyps, diverticular disease and even when no cause is identified [23].

## CONCLUSION

Right‐sided CRC is associated with lower f‐Hb than left. Poorly differentiated and later staged tumours had higher median f‐Hb in this study. These data add to existing evidence suggesting FIT may be less sensitive for right‐sided CRC. Strategies to mitigate the potential for missed or FIT‐negative right‐sided CRC are required, and may include serial or repeat f‐Hb sampling.

## AUTHOR CONTRIBUTIONS

**Nicholas G. Farkas:** Conceptualization; methodology; formal analysis; project administration; writing – original draft; writing – review and editing; data curation. **James O'Brien:** Data curation; writing – original draft; writing – review and editing. **James Norman:** Data curation; writing – review and editing. **Jackie Steinke:** Data curation; writing – review and editing. **Kai Shing Yu:** Formal analysis. **Martin Whyte:** Supervision; writing – review and editing. **Iain Jourdan:** Writing – review and editing; supervision. **Tim Rockall:** Writing – review and editing; supervision. **Sally C. Benton:** Supervision; writing – review and editing; writing – original draft.

## FUNDING INFORMATION

This research received no specific grant from any funding agency in the public, commercial or not‐for‐profit sectors.

## CONFLICT OF INTEREST STATEMENT

The authors declared no potential conflicts of interest with respect to the research, authorship and/or publication of this article.

## ETHICS STATEMENT

No ethical approval was required for this paper.

## Data Availability

The data that support the findings of this study are available from the corresponding author upon reasonable request.
